# Laminar Necrosis and Hypoxic Damage of the Placenta: A Case-Control Study

**DOI:** 10.3390/ijerph19073891

**Published:** 2022-03-24

**Authors:** Katia Mangialardi, Margherita Fanelli, Gerardo Cazzato, Andrea Marzullo, Maria Elisabetta Baldassarre, Antonella Vimercati, Leonardo Resta

**Affiliations:** 1 Department of Emergency and Organ Transplantation (DETO), Section of Pathology, School of Medicine University of Bari “Aldo Moro”, 70124 Bari, Italy; katia.mangialardi@gmail.com (K.M.); gerardo.cazzato@uniba.it (G.C.); andrea.marzullo@uniba.it (A.M.); leonardo.resta@uniba.it (L.R.); 2 Department of Interdisciplinary Medicine, Medical Statistic, School of Medicine University of Bari “Aldo Moro”, 70124 Bari, Italy; 3 Department of Biomedical Sciences and Human Oncology, Neonatology and Neonatal Intensive Care Unit, School of Medicine, University of Bari “Aldo Moro”, 70124 Bari, Italy; mariaelisabetta.baldassarre@uniba.it; 4 Department of Biomedical Sciences and Human Oncology, Gynecologic and Obstetrics Clinic, School of Medicine, University of Bari “Aldo Moro”, 70124 Bari, Italy; antonella.vimercati@uniba.it

**Keywords:** placenta, laminar necrosis, placental dysfunction, placental lesions, hypoxic damage, pregnancy outcome

## Abstract

The aim of this study is to verify the role of laminar necrosis (LN) in the diagnosis of hypoxic damage of the placenta. This is a retrospective case-control study in which 50 cases with laminar necrosis were compared with 100 gestational age-matched controls without laminar necrosis in a 1:2 ratio. The parameters analyzed were: the presence of other placental lesions, obstetric characteristics and neonatal outcome. For each of the 50 cases, the area affected by the lesion was detected, and the lesions were classified into three groups based on the morphology and time of onset of the lesion in order to understand whether these characteristics of the lesion had a clinical-pathology. The results showed that including the search for LN among placental lesions generally examined is useful to guide the pathologist in the diagnosis of placental dysfunction of hypoxic origin.

## 1. Introduction

Hypoxia is an important cause of perinatal morbidity and mortality and can be evaluated retrospectively to explain perinatal outcomes, to assess the risk of relapse in subsequent pregnancies and for medical–legal investigations. However, a hypoxic insult does not always correspond to fetal suffering because the placenta has great compensatory capacities. The histological study of placental lesions allows for reconstruction of the course of pregnancy with the possibility of not only explaining unfavorable outcomes but also identifying damage that is not clinically evident in the mother and fetus. Unique and precise definitions, such as those provided by the Amsterdam International Consensus (a 2014 classification unifying the nomenclature of placental pathology currently used by all pathologists in research and diagnostic practice) [1], help pathologists to correctly diagnose placental lesions [2] and are otherwise often underreported [3]. Laminar necrosis (LN) is one of these elemental lesions of the placenta that has not yet been defined.

Laminar necrosis is a histological feature identified for the first time by Salafia [4] and later more widely studied by Stanek [5,6,7,8] and Goldenberg [9]. It is a non-inflammatory and non-fibrinoid demarcated band of coagulative necrosis involving membranes and the maternal floor. The morphology of laminar necrosis traces the evolution of the lesion [8]. St the beginning, the area of necrosis is characterized exclusively by ghost cells (necrotic cells without a nucleus and persistence of cellular membrane). Subsequently, granulocyte infiltrate becomes gradually more massive and then prevalent with the deposition of karyorrhectic debris (nuclear debris secondary to necrosis).

However, some authors believe that this placental lesion is not a useful marker of hypoxia and that it is necessary to have a better understanding of its significance [10,11]. In the literature of 2021, LN has more often been described as a histological finding of placentas positive for Severe Acute Respiratory Syndrome due to Coronavirus 2 (SARS-Cov-2) [12,13,14,15]. This confirms that LN is arousing more and more interest among pathologists. However, in our opinion, it is not always used correctly; in some cases, it is not really LN but an inflammatory focus of chronic deciduitis (aspecific chronic inflammation of decidua) in the placental basal plate, and LN is a non-inflammatory lesion.

The aim of this case-control study is to further investigate the hypoxic nature of LN, contributing to the results already obtained by Stanek [5,6,7,10] and Goldenberg [8] to provide a precise definition and morphological characterization that can help to recognize LN with a single interpretation and to highlight the possible clinical–pathological associations with laminar necrosis. Furthermore, as it has not yet been studied, in this study, we examine how the time of lesion onset and the area affected by laminar necrosis can influence pregnancy and fetal outcomes. In fact, the lesion was identified not only on membranes and on retroplacental decidua, as reported in the literature but also on disc margin, implant site and capsular decidua.

## 2. Materials and Methods

This is a case-control study in which 50 cases with laminar necrosis at every gestational age were compared with 100 gestational age-matched controls without laminar necrosis in a 1:2 ratio. Cases and controls were recovered from the informatic archive of the Department of Pathology, covering all 1724 placentas sent to pathology for examination between 1 January 2018 and 1 February 2021. All the cases of LN were enrolled in the study group. To select controls, all placentas without laminar necrosis were stratified according to gestational age, and in each stratum, controls were randomly selected, respecting the 1:2 proportion. 

After delivery, all placentas that meet specific and codified criteria approved by Società Italiana di Anatomia Patologica e Citodiagnostica (SIAPeC) (Italian Society of Anatomic Pathology and Diagnostic Cytopathology) are sent for histological examination [16]. This explains why only 13% of cases belonged to a physiological pregnancy and, even among controls, 45% of placentas had signs of hypoxia.

The sampling was carried out according to the standard protocol of the Italian Group of Anatomic Pathology of Embryo Fetus and Appendages (APEFA). The placentas were carefully macroscopically analyzed and then sectioned to obtain at least 2 sections of the umbilical cord, 2 sections of membrane rolls and 3 paracentral full-thickness chorionic disc sections. Additional sections were taken if areas of necrosis, hemorrhage or infarction were identified. Then, the samples were fixed in neutral buffered formalin (10%), dehydrated, paraffin embedded, cut into 4–5 µ slices, deparaffined and stained with hematoxylin-eosin (H & E). The slides were viewed by two pathologists expert in fetal–placental pathology, and in the event of disagreement between the two, the opinion of a third pathologist was sought.

A dataset with pathological and clinical data for cases and controls was created. For each of the 50 cases, the sample area involved by the lesion was detected: membranes, disc margin, retroplacental decidua, implant site and capsular decidua. Lesions were classified into three groups based on morphology and time of lesion onset: lesions with predominantly ghost cells (young lesion), lesions with predominantly leukocyte infiltrate and karyorrhectic debris (old lesion) and lesions with both of (intermediate lesion).

The placental lesions considered were villous infarction, intervillous hemorrhage, retroplacental hematoma, uteroplacental artery atherosis/thrombosis, chorangiosis, chronic villitis of unknown etiology, accelerated villous maturation AVM (increased syncytial knots, accumulation of intervillous fibrin and foci of villous agglutination), distal villous hypoplasia, defective trophoblast in the implantation site, irregular and abnormally shaped hydropic villi and chorioamnionitis.

Obstetric characteristics were detected from the pathology report, including maternal age, gestational age, presence of hypertensive disorders (hypertension, preeclampsia and HELLP syndrome), diabetes, twin pregnancy, changes during labor (non-reassuring or pathological cardiotocographic pattern and emergency cesarean section), intrauterine death, intrauterine growth retardation (IUGR), disorders of amniotic fluid and membranes (oligohydramnios, anhydramnios, polyhydramnios and premature rupture of membranes PROM), placenta previa, abruptio placentae and positivity for SARS-CoV-2/COVID-19 (COronaVIrus Disease 19).

The following information about newborn outcomes was collected from neonatology and the neonatal intensive care unit: days of hospitalization, weight for gestational age (normal for gestational age (NGA), small for gestational age (SGA) or large for gestational age (LGA)), Apgar score at 1 min and at 5 min, neonatal intensive care unit admission, resuscitation, ventilation and intubation, anemia and blood transfusion, acute respiratory distress syndrome (ARDS), intraventricular hemorrhage (IVH), sepsis, necrotizing enterocolitis (NEC), phototherapy, retinopathy of prematurity (ROP) and neonatal death. 

To understand whether the site and the time of onset of the lesion had a clinical-pathological significance, the associations with the outcome of pregnancy, with the clinical information of the mother and fetus (obstetric characteristics), with the neonatal outcome and with the other histopathological findings were analyzed by chi-square test or Fisher exact test. Comparisons between study and control groups were performed by t-test and chi-square as appropriate. Logistic regression analysis was performed to identify possible risk factors for hypoxia. The odds ratio was calculated and evaluated as a measure of risk. The statistical analysis was done using SAS version 9.4. (Statistic Analysis Software, Cary, NC, USA).

## 3. Results

Laminar necrosis was present in 50/1724 (2.9%) of the placentas examined between January 2018 and February 2021. 

The placentas with necrosis examined came from 11 full term deliveries, 18 preterm deliveries, 4 intrauterine deaths and 17 miscarriages. None of the placentas were from SARS-CoV-2/COVID-19-positive cases and controls. Thanks to gestational age matching, there were no significant differences in the distribution of the pregnancy outcome between cases and controls (Table 1). Classifying the causes of miscarriage into hypoxic damage (defective implantation and vascular damage) and other causes (chromosome disease syndromes of the embryo, immunological damage and inflammatory damage), it was found that miscarriages from hypoxic damage accounted for 88.24% in the study group versus 51.52% in the control group, and the difference was highly significant (*p* = 0.01).

There were no statistically significant associations between laminar necrosis and obstetric characteristics (Table 1).

Placental findings are reported in Table 2. Placentas with laminar necrosis were significantly associated with retroplacental hematoma (6% vs. 0%, *p* = 0.04), distal villous hypoplasia (42% vs. 19%, *p* < 0.00) and defective trophoblast in the implantation site (28% vs. 11%, *p* = 0.01). LN is inversely associated with histological diagnosis of chorioamnionitis (0%, vs. 13%, *p* = 0.0039). 

The presence of placental findings such as villous infarction, intervillous hemorrhage, retroplacental hematoma, uteroplacental artery atherosis/thrombosis, AVM, distal villous hypoplasia and defective trophoblast in the implantation site led to the diagnosis of hypoxic damage in 90% of the cases of the study group compared with 45% of the cases of the control group (*p* < 0.0001). 

By means of logistic regression models, hypoxia was highly associated with hypertensive disorders (O.R. = 5.8; 95%CI: 1.95–17.36) and the presence of LN (O.R. = 14; 95%CI: 4.92–40.1) but not with diabetes (O.R. = 0.29; 95%CI: 0.05–1.81).

**Figure 1:**
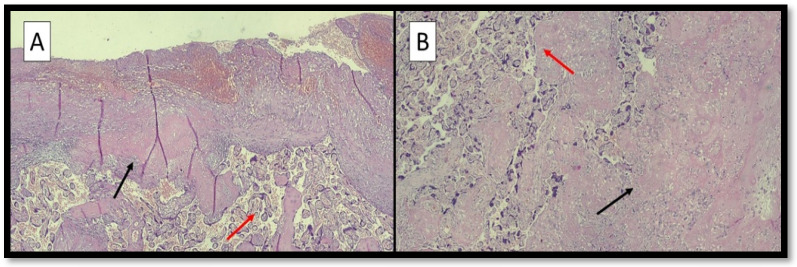
(**A**) Photomicrograph showing peculiar histopathological features associated with hypoxic conditions: villous hypermaturity (red arrow) and Laminar Necrosis (LN) foci (black arrow) (hematoxylin-eosin; original magnification, 4×). (**B**) Photomicrograph showing distal villous hypoplasia (red arrow) and LN foci (black arrow) (hematoxylin-eosin; original magnification, 10×).

Figure 1A,B refers to examples of placental hypoxic findings.

Neonatal clinical findings of 29 newborns from the study group and 63 newborns from the control group are reported In Table 3. There were no significant differences in neonatal outcomes between the study group and the control group, except for sepsis, which was present in 10% of cases and in none of the controls (*p* = 0.03).

Focusing on 50 placentas of the study group, laminar necrosis was identified on membranes in 15 cases, on the disc margin in 8 cases, on the retroplacental decidua in 19 cases, on the implantation site in 9 cases and on the capsular decidua in 2 cases. In some cases, laminar necrosis was present in two sites.

**Figure 2:**
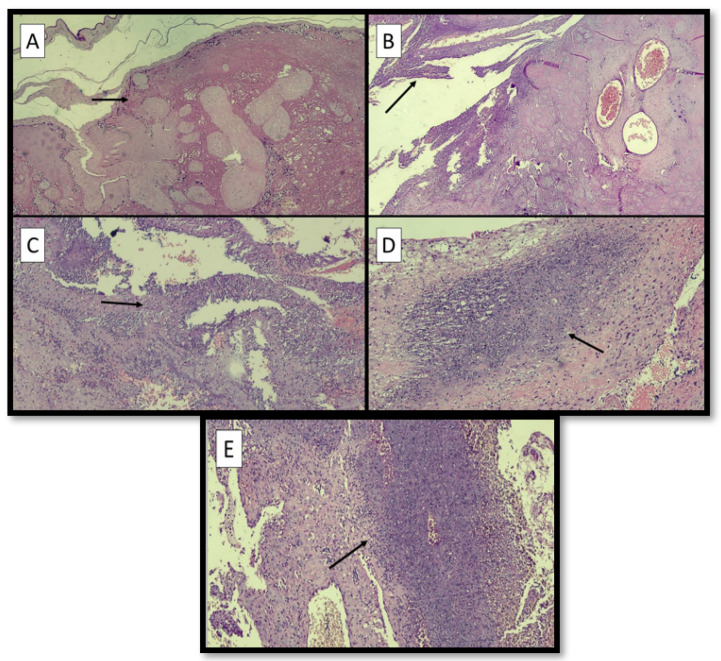
(**A**) Example of LN at the site of membranes (black arrow) (hematoxylin-eosin; original magnification, 4×). (**B**) Example of LN at the margin of disc (black arrow) (hematoxylin-eosin; original magnification, 4×). (**C**) Example of LN at the retroplacental decidua (black arrow) (hematoxylin-eosin; original magnification, 4×). (**D**) Microphotograph of LN at the implantation site (black arrow) (hematoxylin-eosin; original magnification, 10×). (**E**) Microphotograph of LN at the capsular decidua (black arrow) (hematoxylin-eosin; original magnification, 10×).

Figure 2A–E refers to examples of different sites of LN.

In 10 cases, the laminar necrosis was characterized exclusively by ghost cells (young lesion); in 15 cases, both ghost cells and leukocyte infiltrate were present (intermediate lesion; and in 24 cases, leukocyte infiltrate with debris deposition was prevalent (old lesion) *(*Table 4).

**Figure 3:**
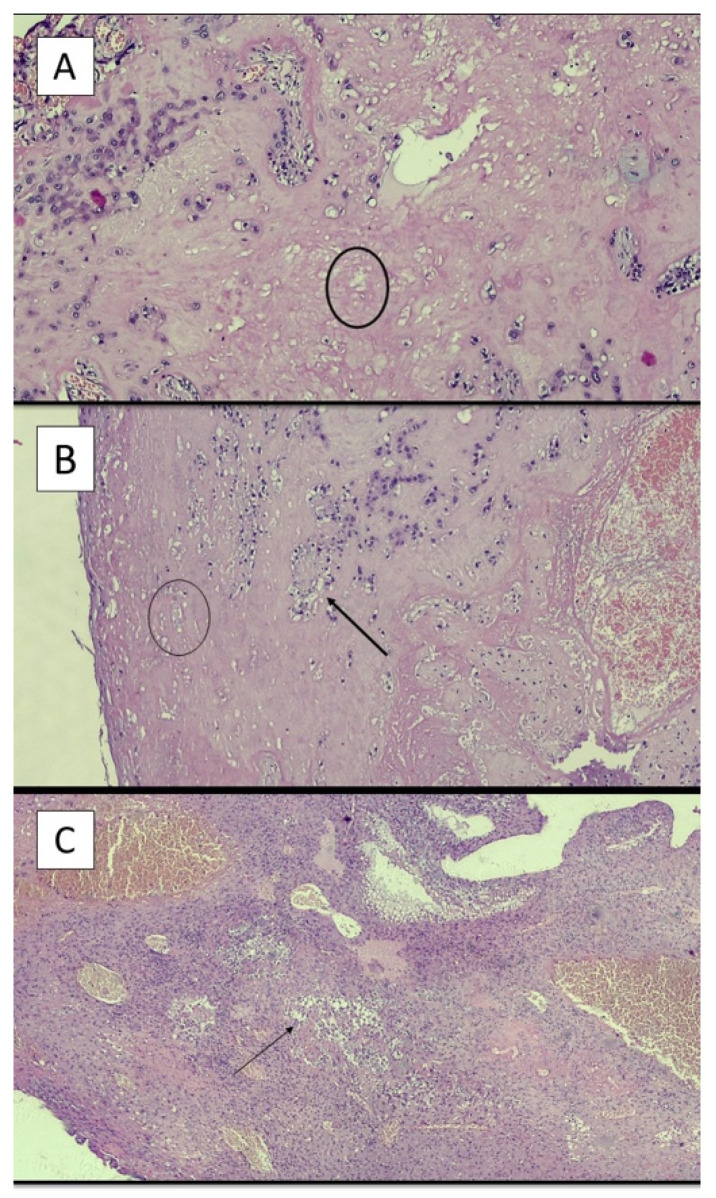
(**A**) Example of young lesion with presence of ghost cells (black circle) (hematoxylin-eosin; original magnification, 4×). (**B**) Example of intermediate lesion with presence of ghost cells (black circle) and neutrophils (black arrow) (hematoxylin-eosin; original magnification, 4×). (**C**) Example of old lesion with a great number of neutrophilic granulocytes (black arrow) (hematoxylin-eosin; original magnification, 4×).

Figure 3A–C refers to examples of different time of onset of LN.

**Figure 4:**
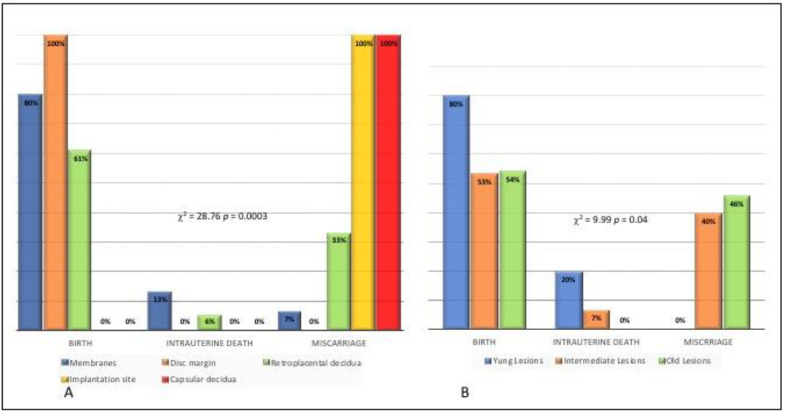
Pregnancy outcome in relation to site of laminar necrosis (**A**) and time of onset of laminar necrosis (**B**).

Figure 4 A,B refers to Pregnancy outcome in relation to site of laminar necrosis (A) and time of onset of laminar necrosis (B).

Pregnancy outcome was significantly associated with the site of the lesion (*p* < 0.001). 

Birth as an outcome was observed in 80% of cases with LN on membranes, in 100% of cases with LN on the disc margin and in 61% of cases with LN on retroplacental decidua. No birth as outcome was observed when LN was at the implantation site or capsular decidua. In contrast, miscarriages were observed in 100% of cases with LN at the site implantation, in 100% of cases with LN on capsular decidua, in 33% of cases with LN on retroplacental decidua and in only one case (7%) with LN on the membrane (Figure 4A).

A significant association was found between pregnancy outcome and time of onset of the lesion (*p* = 0.04). Birth was the outcome observed in 80% of cases presenting placentas with young lesions, in 53% of cases with intermediate lesions and in 54% of cases with old lesions. Intrauterine death was observed in none of the placentas with old lesions, in 20% placentas with young lesions and 7% placentas with intermediate lesions. Miscarriage was observed in no cases of placentas with young lesions, in 40% with intermediate lesions and in 46% with old lesions (Figure 4B). To confirm that old lesions are most frequent in cases of miscarriage, it was observed that 33% of old lesions are located on the implantation site vs. 7% of intermediate lesions and 0% of young lesions (*p* = 0.03). It should be noted that 100% of lesions at the implantation site are associated with miscarriage (Figure 4A).

Among obstetric characteristics, only IUGR was significantly associated with the site of laminar necrosis. IUGR was observed in 40% of cases with LN on the membrane vs. 14.7% of cases with LN on a different site (*p* = 0.05) (O.R. = 3.87; 95%CI: 0.95–15.7). IUGR was observed in 62.5% of cases with LN on the margin of disc vs. 14.6% of cases with LN on a different site (*p* < 0.01) (O.R. = 0.7; 95%CI: 1.82–51.8).

No association between the site of the lesion and neonatal outcome was observed (*p* > 0.05).

The time of onset of the lesions was not associated with obstetric characteristics (*p* > 0.05) or with neonatal outcome (*p* > 0.05).

Early and late maternal malperfusion were diagnosed in the presence of villous hypoplasia and distal villous hypermaturity (accellerated villous maturation), respectively. Among the 30 cases in which maternal malperfusion was diagnosed, a strong association between early or late malperfusion and time of onset of the lesions was found. A total of 35% of early malperfusions presented young laminar necrosis vs. 22.2% of late malperfusions. Intermediate lesions were not present in any cases of late malperfusion vs. 40% of early malperfusion cases. A total of 77% of late malperfusions presented old lesions vs. 25% of early malperfusions (*p* = 0.017).

## 4. Discussion

This study allowed us to shed light on the pathogenetic nature of laminar necrosis thanks to the large amount of material examined. The ongoing debate on the hypoxic origin of lesions is particularly complicated because the origin is difficult to determine. In our analysis, LN was present in only 2.9% of the material examined. This is contrary to the report of Bendon et al. [10], who found that LN is a very common lesion, involving 28% of all placentas. In order to reach a diagnosis of LN, it is necessary to exclude many features that simulate it, such as, a recent post-detachment lysis, microhemorrhages, inflammatory foci of chronic deciduitis in the placental basal plate and non-specific inflammatory infiltrates (the latter are not demarcated and therefore cannot be considered reliable). If all these lesions without necrotic–ischemic significance are excluded, true LN is extremely rare. 

A first interesting result is the statistically significant association of LN with retroplacental hematoma, distal villous hypoplasia and defective trophoblast in the implantation site, which are all features of maternal vascular malperfusion (Table 2). Retroplacental hematoma is the result of untimely detachment of the placenta. It appears as a blood clot adherent to the basal plate, and the parenchyma overlying the hematoma is excluded from oxygenation. Distal villous hypoplasia is defined by areas with decreased numbers of thin and poorly branching villi [17]. Defective trophoblast in the implantation site configures a picture of shallow placentation. 

Confirming the non-inflammatory nature of LN, placentas with LN were less likely to have a histological diagnosis of chorioamnionitis than the control group (13% vs. 0%). This was previously reported by Goldenberg et al. [9], who observed an inverse association between LN and placental histological markers of acute inflammation; positive placenta cultures; and high cord IL-6 levels, a recognized marker of intrauterine infection. 

Analyzing the neonatal outcomes, LN was statistically associated with sepsis. This, at first glance, might seem contradictory, but in reality, strong risk factors for development of neonatal sepsis there include prematurity, low birth weight and low Apgar score at birth [18], which are common in the case of hypoxia.

By means of logistic regression models, it emerged that the presence of LN increases the possibility of diagnosing hypoxic damage by 14 times (O.R. = 14; 95%CI: 1.73–13.43). The link between these two variables was found to be even greater than that existing between hypoxia and hypertensive disorders (O.R. = 4.8, 95%CI: 1.73–13.43). This is diriment evidence for the interpretation of LN.

Once the hypoxic nature of the lesion was demonstrated, it was then possible to observe that the site and the time of onset of the LN has a clinical significance: it affects the outcome of pregnancy. Cases leading to birth presented placentas with LN on membranes, disc margin, and retroplacental decidua but never at the implantation site or capsular decidua; these latter sites are instead associated with miscarriage (100%). This can be explained by the fact that the implantation site is an area primarily involved and decisive in the placentation process; therefore, hypoxic stress in this area is more severe and can lead to death, and the capsular decidua is an area of strong oxidative stress. 

Hypoxic suffering involving less crucial sites can be compensated for because “placental oxygen consumption is four times higher than fetal oxygen consumption” [8].

Furthermore, placentas from cases of birth mainly present young lesions with shadow cells, whereas placentas from miscarriages present old lesions with granulocytic infiltrates. The obtained results are in agreement with what is expected, as the timing of hypoxic–ischemic intrauterine stress affects the extent of damage, and once the fetus has died, the increase in granulocyte infiltrate is explained by the permanence of the placenta in the maternal uterus.

Considering only the cases that led to birth despite malperfusion, old lesions are much more frequent in cases of late malperfusion than in cases of early malperfusion. Maternal malperfusion that arises early is a less serious malperfusion with a slower progress; otherwise, precisely because it arises at such a precocious and delicate phase, it would cause the death of the fetus. On the contrary, late malperfusion has a greater extension and more rapid and aggressive action, which explains the increased presence of granulocytes and the deposition of karyorrhectic debris. 

Furthermore, if the site of laminar necrosis is the margin of the disc, the risk of the fetus developing IUGR is 8.9 times greater. The involvement of the disc margin could be demonstrate that the laminar necrosis represents the expression of hypoxic suffering, as it is a transition point between the chorionic disc and the membranes with a lower maternal blood supply and therefore a lower compensatory capacity (watershed infarct), as happens in threatened miscarriages.

## 5. Conclusions

Our analysis is part of a lively and ongoing debate. It was therefore our goal to clarify the nature and significance of LN by using a large sample from which to obtain reliable results.

This study shows that LN represents an important histological finding; therefore, in our opinion, it must be evaluated in combination with other findings to reach a correct diagnosis of hypoxic damage. Furthermore, the results obtained suggest that it is also important to evaluate the site of LN and the time to onset, as they seem to have a relevant clinical impact and have not yet been investigated in the literature.

## Figures and Tables

**Table 1:** Obstetric characteristics in study and control group.

Obstetric Characteristics	Study Group (50 Cases)	Control Group (100 Cases)	*p*-Value
Maternal age	34.18 ± 5.99	34.49 ± 5.92	0.76
Gestational age at delivery	26.24 ± 12.36	26.32 ± 12.33	0.97
Hypertensive disorders, *n* (%)	9 (18%)	22 (22%)	0.57
Diabetes, *n* (%)	3 (6%)	4 (4%)	0.43
Twin pregnancy, *n* (%)	3 (6%)	11 (11%)	0.91
Changes during labor, *n* (%)	14 (28%)	24 (24%)	0.60
Intrauterine growth restriction, *n* (%)	11 (22%)	13 (13%)	0.16
Disorders of amniotic fluid and membranes, *n* (%)	8 (16%)	15 (15%)	0.87
Placenta previa/ abruptio placentae, *n* (%)	3 (6%)	2 (2%)	0.21
Pregnancy outcome			0.12
Birth, *n* (%)	29 (58%)	63 (63%)	
Intrauterine death, *n* (%)	4 (8%)	2 (2%)	
Miscarriage, *n* (%)	17 (34%)	30 (30%)	
Therapeutic termination of pregnancy, *n* (%)	0 (0%)	5 (5%)	
Cesarean section, *n* (%) *	23 (79%)	42 (42%)	0.35
Preterm birth, *n* (%) *	18 (62%)	40 (63%)	0.89

* percentages were calculated on the total of live births.

**Table 2:** Placental findings in study and control groups.

Placental Findings	Study Group (50 Cases)	Control Group (100 Cases)	*p*-Value
Villous infarction, *n* (%)	10 (20%)	10 (10%)	0.09
Intervillous hemorrhage, *n* (%)	9 (18%)	19 (19%)	0.88
Retroplacental hematoma, *n* (%)	3 (6%)	0 (0%)	0.04
Uteroplacental arteries atherosis/thrombosis, *n* (%)	5 (10%)	5 (5%)	0.21
Chorangiosis, *n* (%)	3 (6%)	6 (6%)	0.63
Chronic villitis of unknown etiology, *n* (%)	2 (4%)	7 (7%)	0.37
Accelerated villous maturation (AVM), *n* (%)	9 (18%)	12 (12%)	0.50
Distal villous hypoplasia, *n* (%)	21 (42%)	19 (19%)	0.00
Defective trophoblast in the implantation site, *n* (%)	14 (28%)	11 (11%)	0.01
Irregular and abnormally shaped hydropic villi, *n* (%)	4 (8%)	15 (15%)	0.17
Chorioamnionitis, *n* (%)	0 (0%)	13 (13%)	0.00

**Table 3:** Neonatal findings in study and control group.

Neonatal Clinical Findings	Study Group (29)	Control Group (63 *)	*p*-Value
Neonatal intensive care unit (NICU) admission, *n* (%)	16 (55%)	21 (35%)	0.09
Resuscitation, *n* (%)	10 (34%)	19 (32%)	0.79
Ventilation, *n* (%)	10 (34%)	19 (32%)	0.79
Intubation, *n* (%)	4 (14%)	5 (8%)	0.32
Anemia and blood transfusion, *n* (%)	4 (14%)	5 (8%)	0.32
Acute Respiratory Distress Syndrome (ARDS), *n* (%)	9 (31%)	17 (28%)	0.79
Intraventricular hemorrhage (IVH), *n* (%)	2 (7%)	1 (2%)	0.25
Sepsis, *n* (%)	3 (10%)	0 (0%)	0.03
Necrotizing enterocolitis (NEC), *n* (%)	1 (3.5%)	0 (0%)	0.33
Phototherapy, *n* (%)	7 (24%)	7 (12%)	0.13
Retinopathy of prematurity (ROP), *n* (%)	3 (10%)	3 (5%)	0.30
Neonatal death, *n* (%)	1 (3%)	2 (3%)	0.71
APGAR 1 min < 7	7 (24%)	12 (19%)	0.58
APGAR 5 min < 7	1 (3%)	4 (6%)	0.57
Small for Gestational Age (SGA)	16 (57%)	39 (66%)	0.17

* percentages were calculated on the total of live births (three controls missing). APGAR stands for “Appearance, Pulse, Grimace, Activity, and Respiration”.

**Table 4:** Site and time of onset of the lesion.

**Site of the Lesion**	**Number of Lesions**
Membranes, *n* (%)	15 (30%)
Disc margin, *n* (%)	9 (18%)
Retroplacental decidua, *n* (%)	19 (39%)
Site of implantation, *n* (%)	9 (18%)
Capsular decidua, *n* (%)	2 (4%)
**Time of Onset of the Lesion**	**Number of Lesions**
Young lesion, *n* (%)	10 (20%)
Intermediate lesion, *n* (%)	15 (31%)
Old lesion, *n* (%)	24 (49%)

Note: more than one site is possible. Site and time of onset were evaluated on 49 lesions.
